# COVID-19 Vaccine as a Potential Triggering Factor for Anti-Glomerular Basement Membrane (GBM) Disease: A Case Report and Literature Review

**DOI:** 10.7759/cureus.29075

**Published:** 2022-09-12

**Authors:** Mohanad Ahmed, Sabah Mohamed, Hussein Alhussein, Isra Eltazi, Rayan M Sibira, Ahmad Abdulhadi

**Affiliations:** 1 Rheumatology, Hamad General Hospital, Doha, QAT; 2 Internal Medicine, Hamad General Hospital, Doha, QAT; 3 Neurology, Hamad General Hospital, Doha, QAT; 4 Laboratory Medicine and Pathology, Hamad General Hospital, Doha, QAT

**Keywords:** goodpasture syndrome, glomerulonephritis, crescent shape glomerulonephritis, anti-gbm disease, covid-19 vaccine

## Abstract

Coronavirus 2019 (COVID-19) is considered one of the most significant medical pandemics of this century, with high morbidity and mortality associated with the pandemic. The virus was recognized initially as a cause of pneumonia, but subsequent studies showed significant association with gastrointestinal, neurological, and autoimmune diseases. By 2020, several vaccines became available for use, significantly reducing the infection rate. A good safety profile supported most of the studies related to vaccines. However, this area is still under study, and some reports linked the COVID-19 vaccine to the development of thrombocytopenia, thrombosis, Guillain-Barre syndrome, autoimmune diseases, and myocarditis. These side effects need to be reported to VAERS (Vaccine Adverse Event Reporting System). The exact etiology of anti-glomerular basement (Anti-GBM) disease remains unknown, but the disease is thought to be triggered by environmental factors in genetically predisposed individuals. It is considered one of the serious diseases that could lead to permanent kidney impairment if not treated early and adequately. That's why a great effort is being made by health care practitioners to figure out and avoid the risk and triggering factors. Few previously published papers linked the COVID-19 vaccine and the development of anti-GBM disease, which raised concerns about digging more into this area.

Herein, we are reporting a case of a patient who developed rapidly progressive glomerulonephritis (RPGN) due to anti-glomerular basement membrane (GBM) antibody disease two days after receiving the second dose of the COVID-19 vaccine.

## Introduction

The coronavirus 2019 (COVID-19) pandemic that caused striking worldwide extensive morbidity and mortality during the past few years was followed by heroic research and vaccine discoveries, altering the course of the illness in a more benign direction. The worldwide vaccination campaigns were started quickly and aggressively to tackle the disease's spread. Currently, COVID-19 vaccines have reached billions of people worldwide. The evidence is overwhelming that no matter which one you take, the vaccines offer life-saving protection against a disease that has killed millions. Still, adverse events not previously observed during clinical trials are now sprouting and should remain in focus and be tracked and followed up vigilantly. There has been an upward trend in reporting cases of unmasking/reactivation of glomerular disease after receiving mRNA vaccines.

Antiglomerular basement membrane (anti-GBM) disease is an autoimmune vasculitis disease characterized by the production of autoantibodies against type IV collagen in the basement membrane affecting both kidneys and lungs. These autoantibodies cause capillaritis at the mentioned sites, and patients usually succumb to rapidly progressive glomerulonephritis and alveolar bleeding. Some forms of the disease involve just the lung or the kidney. Anti-GBM disease used to be known as Goodpasture syndrome. Symptoms of the disease include coughing of blood, dry cough, shortness of breath, blood in the urine, burning sensation when urinating and other systemic symptoms like weight loss, loss of appetite, nausea, and vomiting. Diagnosis is made by linking clinical data and serologic testing, though renal biopsy is required for confirmation [1]. Like most autoimmune diseases, anti-GBM disease occurs in genetically predisposed individuals after a specific insult such as an infection, drugs, environmental exposure, etc. [2].

Different vaccines use different mechanisms to generate immunity. For example, Pfizer BNT162b2 and Moderna mRNA-1273 use a pioneer mechanism wherein a lipid nanoparticle nucleoside-modified mRNA encodes SARS-CoV-2 spike (S) protein which prevents host attachment and viral entry. AstraZeneca uses a replication-deficient chimpanzee adenovirus vector containing the SARS-CoV-2 S protein. It has been shown recently that vaccines, specifically mRNA-based ones, are linked to the development of glomerular disease [3].

## Case presentation

We present a 26-year-old male, previously healthy, working as a laborer in a building company. The patient was sent on 16th August 2021 to our medical department from a field hospital complaining of cough, hemoptysis, shortness of breath, and feeling fatigued for seven days which increased in severity in the prior two days. He mentioned no chest pain or fever. On further questioning, he said he received the second dose of the Moderna COVID-19 vaccine on 14th August, while the first was given on 17th July. He doesn’t smoke or consume alcohol and is not taking any medications. On examination, the patient looked in mild respiratory distress. His vital signs showed a respiratory rate of 23 cycles/min, a pulse rate of 98 beats/min, and blood pressure of 115/76 mm Hg. He was afebrile, and his oxygen saturation was 96% on room air. Chest examination was significant for diffuse bilateral crackles with scattered wheezes. Cardiovascular, abdomen and neurologic exams were unremarkable. The patient was started on salbutamol nebulization while blood tests and chest X-rays were requested.

**Figure 1:**
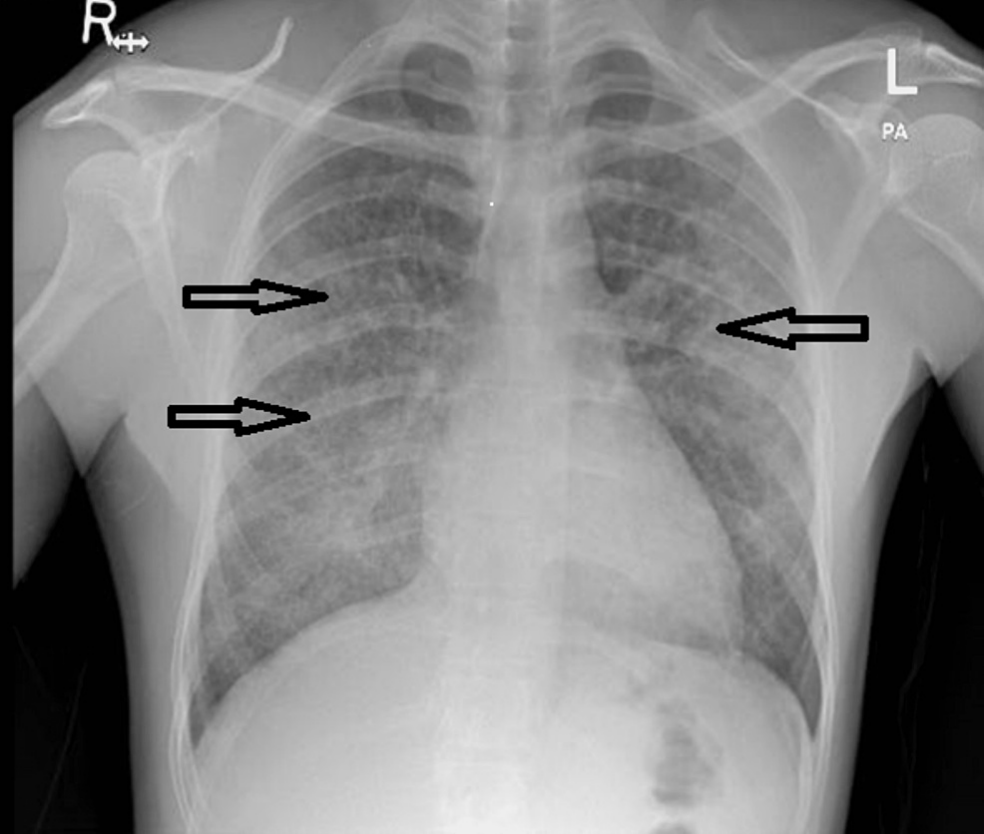
Chest X-ray on initial presentation showed bilateral diffuse airspace opacity and patchy consolidative changes at both lung parenchyma.

Initial lab results were significant for a hemoglobin (Hb) of 5.6 gm/dl (normal: 13 to 15 gm/dl); platelets count was 231,000/mcl (normal: 150,000 to 400,000 mcl); blood urea was 15 mmol/l (normal: 2.1 to 8.5 mmol/L); creatinine was 641 µmol/L (normal: 61.9 to 114.9 µmol/L); potassium was 4.1 mmol/l (normal: 3.5 to 5.3 mmol/L), and bicarbonate was 18 mmol/l (normal: 18 to 24 mmol/L). Chest X-ray showed bilateral diffuse airspace opacity and patchy consolidative changes at both lung parenchyma with background lung nodular shadow (Figure 1).

Initial management included transfusion of two units of packed red blood cells and started on Ceftriaxone plus azithromycin antibiotics to cover the possibility of community-acquired pneumonia while the admission process was initiated.

**Figure 2:**
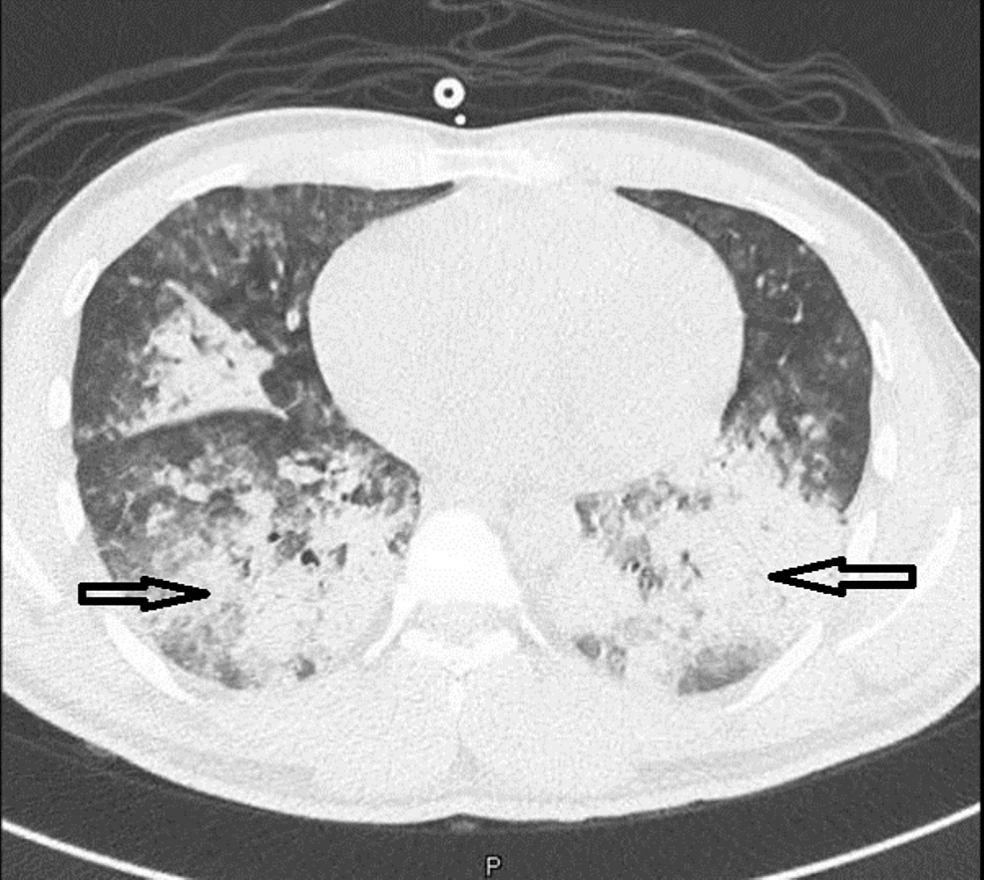
CT of the chest showing bilateral diffuse consolidation with centrilobular nodules, in keeping with alveolar space disease.

Ultrasound scan of both kidneys showed normal size (right kidney measures 11 x 4.9 cm, left kidney measures 11.5 x 6.8 cm) and no evidence of obstruction. The next day, the patient's laboratory tests showed worsening urea and creatinine, and he started to feel more shortness of breath. Computed tomography (CT) scan of the chest was done (Figure 2), followed by bedside bronchoscopy, and the presence of pulmonary hemorrhage was confirmed.

The patient was started on pulse steroids (a dose of 1000 mg of methylprednisolone daily for five days). Serology reports of the antinuclear antibody (ANA) and antineutrophil cytoplasmic antibody (ANCA) were negative, and an anti-glomerular basement membrane antibodies test was sent. On 19th August 2021, echocardiography was done and reported as normal, followed by an ultrasound-guided kidney biopsy on the same day.

Although the patient was started on steroids, he continued to deteriorate. His oxygen requirement increased, kidney function parameters (urea and creatinine) continued to decline, and he developed lower limb edema, followed by anuria. At this point, the patient was transferred to the intensive care unit, a central line was inserted, and the nephrology team started him on intermittent hemodialysis. Four days later, the report of anti-GBM antibody was released. It was positive with a titer of 550.0 U/mL (normal: less than 7 U/mL). The nephrology team decided to start plasma exchange. A course of daily plasma exchange was done in the first week, followed by plasma exchange every other day in the second week. Weekly measurement of the antibody titer showed continuous decline till it became negative. In addition, the patient was started on oral Cyclophosphamide 100 mg daily, and the plan was to continue for three months. On 23rd August, the biopsy result was reported as a crescentic glomerulonephritis anti-GBM disease pattern.

Pathological finding

**Figure 3:**
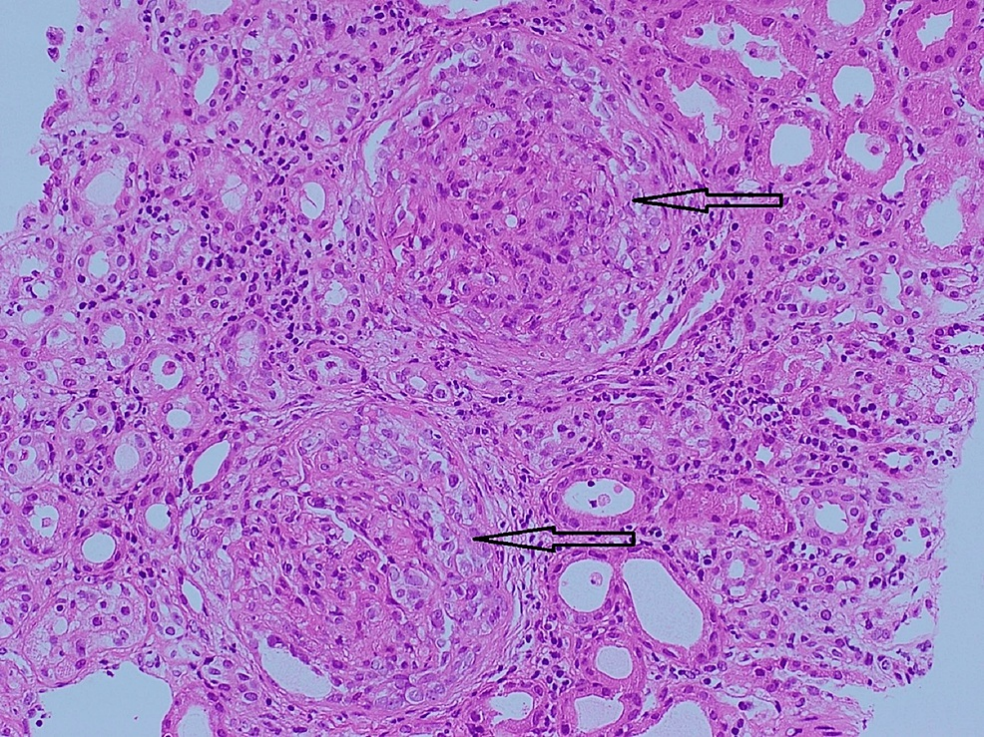
Uniform cellular crescents with periglomerular inflammation (H&E, x200).

**Figure 4:**
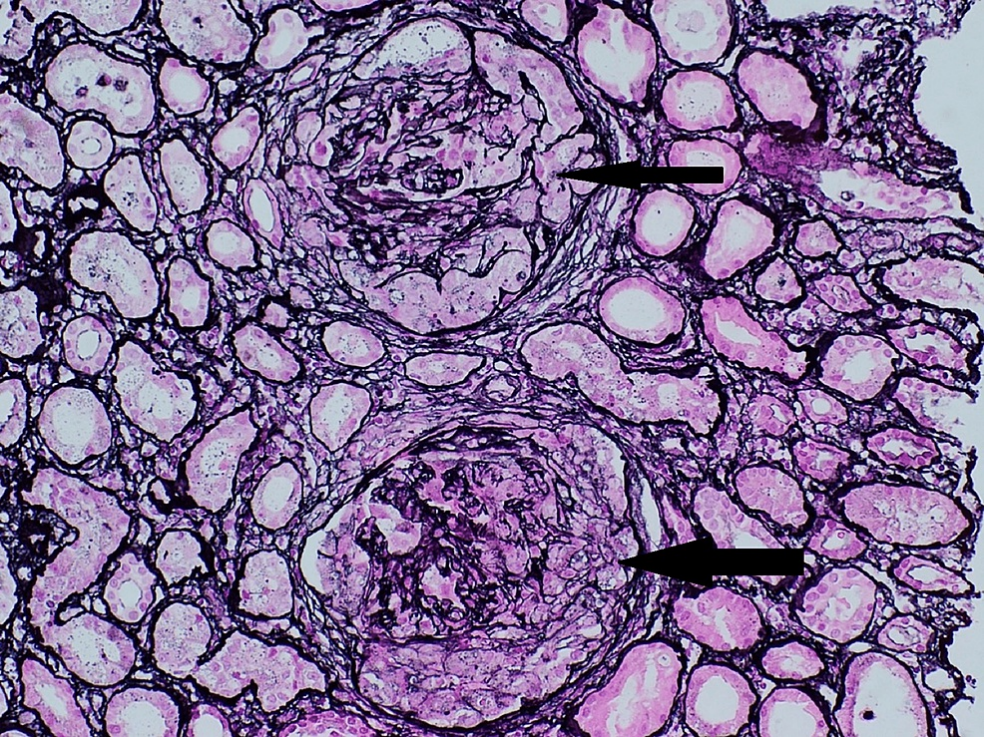
Remnants of GBM surrounded by cellular crescents, which fill the space delineated by Bowman's capsule (Jones silver stain, x200). GBM: Glomerular Basement Membrane

**Figure 5:**
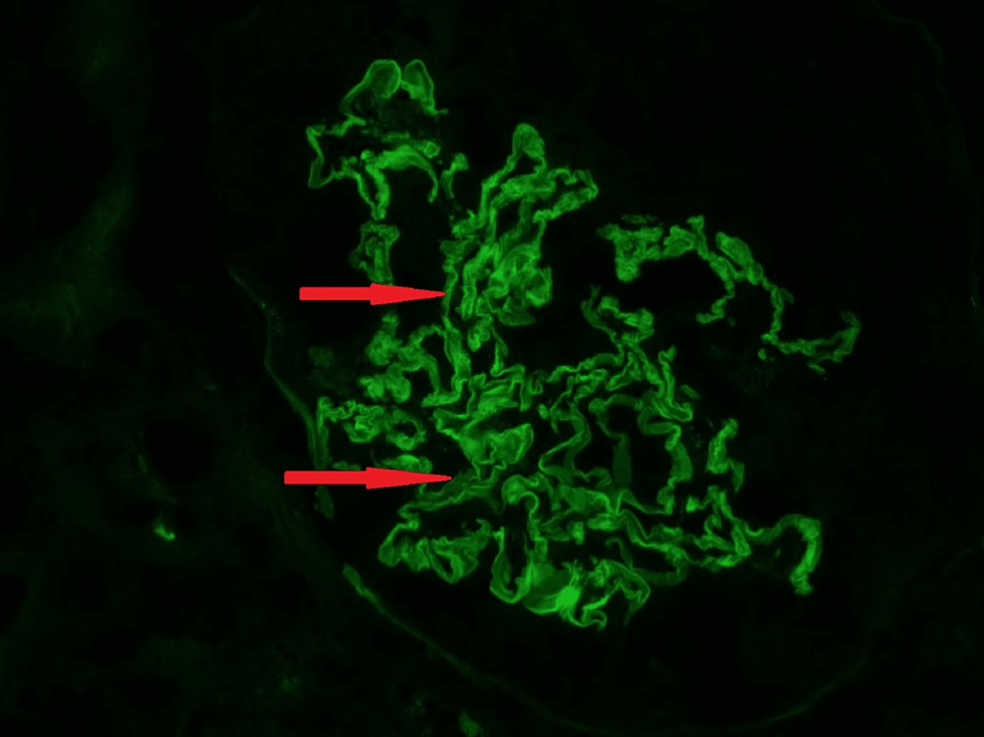
Strong linear ribbon-like appearance IgG (2+) in the GBM, the glomerulus is compressed by a crescent, which does not stain (Direct immunofluorescence on frozen kidney tissue, x200). GBM: Glomerular Basement Membrane

Kidney biopsy (Figures 3-5) showed 24 glomeruli, and 23 glomeruli showed extra capillary cellular crescents, but none showed fibro cellular/fibrous crescents. The crescents are temporally homogenous at the same age and stage and fill the space delineated by Bowman's capsule (Figures 3, 4), and some show rupture of Bowman's capsule. Some glomeruli show active periglomerular inflammation (Figure 3). Mild tubular atrophy and interstitial fibrosis were also noted (10-15% of the cortical tissue), associated with moderate multifocal interstitial inflammatory infiltrate composed of lymphocytes and occasional eosinophils. Direct immunofluorescent microscopy showed linear staining of IgG (Figure 5), complement component C3, and light chains (kappa and lambda) along the glomerular basement membrane. These findings of crescentic glomerulonephritis are consistent with anti-glomerular basement membrane (GBM) glomerulonephritis.

The patient had a total of 10 plasma exchange sessions. Hemodialysis was daily in the first week, every other day in the second week, and then switched stop with a plan to do sessions on need only. Cyclophosphamide continued, while oral steroids started with 40 mg prednisolone daily with a slowly tapering regimen after five days of intravenous pulse steroids.

A few days later, the patient showed significant improvement in his clinical condition and laboratory tests. His kidney function improved, symptoms resolved, and hemodialysis was stopped. The weekly titer of the anti-GBM antibody was steadily declining with plasmapheresis. After three weeks, the measured titer dropped from 500 U/mL to 7 U/mL and was maintained on oral prednisolone 40 mg daily and Cyclophosphamide 100 mg daily.

## Discussion

Since the start of the COVID-19 vaccination, the temporal association between the vaccine and the development of de novo or relapse of glomerular diseases, including anti-GBM, has been studied (Table 1) [4]. However, the pathogenesis of vaccine-associated glomerular disorders has not been fully elucidated. On reviewing the literature, we found a very small number of reported cases of de novo anti-GBM disease following the COVID-19 vaccine [5-7].

**Table 1:** Cases of de novo anti-GBM disease post-mRNA COVID-19 vaccines S: Steroid, PLX: Plasmapheresis, CYP: Cyclophosphamide, O: Oral, IV: Intravenous, AKI: Acute kidney injury, NRP: Nephrotic range proteinuria.

	Authors	Report	Age/gender	Type of vaccine	Dose	Days from vaccine to onset	De novo or relapse	Symptoms on presentation	ANCA association	Treatment	Other triggers	Outcome
1	Nagai et al. [5]	Japan	F, 70 YO	N/A	2^nd^	9 days	De Novo	AKI, Hematuria	-ve	S, PLX, IV CYP	Centipede bite	Remission
2	Sacker et al. [7]	USA	F, older	Moderna	2^nd^	14 days	De Novo	AKI, hematuria	-ve	S, PLX, CYP	N/A	HD dependent
3	Tan et al. [6]	Singapore	F, 60 YO	Pfizer	2^nd^	1 day	De Novo	AKI, Hematuria, NRP	-ve	S, PLX, O CYP	N/A	N/A
4	Our case	Qatar	M, 26 YO	Moderna	2^nd^	2 days	De Novo	Hemoptysis, AKI	-ve	S, PLX, O CYP	N/A	HD dependent

Anti-GBM is a rare disease with a bimodal distribution. The majority of the young patients present with pulmonary manifestations, while most of the elderly patients present with renal involvement [8,9]. Comparing our patient to the previously reported cases, all reported cases were elderly females who presented with hematuria and acute kidney injury following the COVID-19 vaccine. However, our patient was a young male who presented with symptomatic pulmonary hemorrhage and renal impairment simultaneously.

The duration between exposure to the vaccine and the incidence of anti-GBM disease varied between days to weeks [5-7]. While the symptoms started two days after the second dose of Moderna vaccine in our reported patient. Although we were unable to find other triggers, they cannot be excluded entirely. All reported cases, including our patient, showed a negative association with antineutrophil cytoplasmic antibodies (ANCA). In all patients, the symptoms started after the second dose of the vaccine, and they were all asymptomatic after the first dose.

The outcome of the reported cases showed no specific pattern. Despite receiving steroid cyclophosphamide and plasmapheresis, our patient's renal function did not improve, and he remained dialysis-dependent.

## Conclusions

Our case report and literature review of the previously published cases may help offer insight into the consequences course of the COVID-19 vaccine. We report a case of a patient who presented with anti-GBM disease following the COVID-19 vaccine and who was previously healthy before that. This raises the possibility of establishing a direct causal association between the COVID-19 vaccine and anti-GBM disease. However, future reports and extensive epidemiological studies are needed to ascertain the nature of this relationship. Moreover, physicians should have high clinical suspicions when encountering patients with anti-GBM disease following the COVID-19 vaccine.
